# Identification of a metabolomic signature associated with feed efficiency in beef cattle

**DOI:** 10.1186/s12864-018-5406-2

**Published:** 2019-01-07

**Authors:** Francisco José Novais, Pedro Ratto Lisboa Pires, Pâmela Almeida Alexandre, Robert A Dromms, Amadeu Hoshi Iglesias, José Bento Sterman Ferraz, Mark Philip-Walter Styczynski, Heidge Fukumasu

**Affiliations:** 1 Department of Veterinary Medicine, School of Animal Science and Food Engineering, University of Sao Paulo, Av. Duque de Caxias Norte n°225, Pirassununga, 13635-900 Sao Paulo Brazil; 2 School of Chemical & Biomolecular Engineering, Georgia Institute of Technology, Atlanta, Georgia USA; 3 Apex Science, Campinas, Sao Paulo Brazil

**Keywords:** Residual feed intake, Nellore, Retinol, WGCNA

## Abstract

**Background:**

Ruminants play a great role in sustainable livestock since they transform pastures, silage, and crop residues into high-quality human food (i.e. milk and beef). Animals with better ability to convert food into animal protein, measured as a trait called feed efficiency (FE), also produce less manure and greenhouse gas per kilogram of produced meat. Thus, the identification of high feed efficiency cattle is important for sustainable nutritional management. Our aim was to evaluate the potential of serum metabolites to identify FE of beef cattle before they enter the feedlot.

**Results:**

A total of 3598 and 4210 m/z features was detected in negative and positive ionization modes via liquid chromatography-mass spectrometry. A single feature was different between high and low FE groups. Network analysis (WGCNA) yielded the detection of 19 and 20 network modules of highly correlated features in negative and positive mode respectively, and 1 module of each acquisition mode was associated with RFI (*r* = 0.55, *P* < 0.05). Pathway enrichment analysis (Mummichog) yielded the Retinol metabolism pathway associated with feed efficiency in beef cattle in our conditions.

**Conclusion:**

Altogether, these findings demonstrate the existence of a serum-based metabolomic signature associated with feed efficiency in beef cattle before they enter the feedlot. We are now working to validate the use of metabolites for identification of feed efficient animals for sustainable nutritional management.

**Electronic supplementary material:**

The online version of this article (10.1186/s12864-018-5406-2) contains supplementary material, which is available to authorized users.

## Background

The Food and Agriculture Organization of the United Nations estimates the world population will reach 9 billion people by 2050 and as a consequence, livestock production must double to meet the demand for food [1]. Sustainable livestock production is a field of intense research where ruminants play a great role since they can transform graze pastures, silage and high-fiber crop residues into high-quality human food (i.e. milk and meat) [2]. The goal is “sustainable intensification” [3], meaning increased productivity while reducing the environmental impacts. In this context, feed efficiency (FE) has a particular importance, since it is directly related to productivity, greenhouse gas emission intensities, and resource use [4, 5].

Due to its importance, more than two dozen feed efficiency measurements have been proposed to select efficient animals and from those, residual feed intake (RFI) is considered one of the most effective methods [6, 7]. As a complex trait, at least five major physiological mechanisms contribute to RFI variation: feed intake behavior, digestion, physical activity, thermoregulation and cell anabolism/catabolism [8]. Recently, our group proposed a new biological process associated with FE in beef cattle: increased hepatic inflammation in less efficient animals probably caused by altered lipid metabolism and/or increased bacterial infection associated with higher feed intake [9].

Metabolomics is the systems-scale study of low-molecular-weight biochemicals (< 1500 Da) involved in metabolism, including carbohydrates, lipids, amino acids, biogenic amines, and organic acids [10, 11]. Due to the important role of metabolism across all biological processes, metabolomics studies have been increasingly used to understand physiological processes associated with economically important traits in livestock such as meat quality in pigs [12], milk production in dairy cattle [13–15] and growth in beef cattle [16]. Also, metabolomics has been applied to RFI studies, reporting blood metabolites in beef cattle during feedlot [17, 18].

Currently, there is an urgent need to develop new ways to predict FE in livestock, since the use of the commercially available genomic markers for genetic selection is not sensitive enough due to low to moderate heritability (ranging from 0.08 to 0.49) of the FE trait [19–22]. Therefore, we hypothesized there are specific serum metabolome signatures that predict feed efficiency in beef cattle before the feedlot which could be used for feed management of beef cattle. To this end, we used serum samples from a previous feeding trial with young Nellore bulls and performed a metabolomic approach on high and low feed efficient animals. The resulting data were used to investigate whether circulating metabolite levels could predict feed efficiency.

## Methods

### Phenotypic data collection

All animal procedures were approved by the Institutional Animal Care and Use Committee of the Faculty of Food Engineering and Animal Science at the University of Sao Paulo (protocol 14.1.636.74.1). The serum of 98 Nellore young bulls (16 to 20 months old and 376 ± 29 kg BW) born and raised in the University of Sao Paulo were collected 21 days prior to a 70-d feedlot. Briefly, the feeding-trial period was preceded by 21 days of adaptation to diet and location and before that, the animals were maintained in a single group on *Brachiaria spp.* pastures. On adaptation period, animals received corn silage (ad libitum), gradually replaced by trial diet (total mixed ration, including dry corn grain, corn silage, soybean, citrus pulp pellets, urea, calcareous, mineral salt and potassium chloride) offered at 8:00 h and 16:00 h. After the experiment, all animals were slaughtered following the guidelines of the Institutional Animal Care and Use Committee. More details regarding animals, diet and experimental design can be found in Alexandre et al. [9] and Mota et al. [23].

RFI was calculated as the difference between the expected and observed feed intake, considering the average metabolic weight (MBW) and ADG to predict DMI [6]. The 98 animals were ranked by RFI, and two groups of 8 animals each were selected for further analysis (total of 16 animals): high feed efficiency (HFE, low RFI) and low feed efficiency (LFE, high RFI). Sire and age effect on RFI were estimated by completely randomized design on linear model:

$$ Yijk=\mu +\beta i+\beta k+ eij $$

where *Yij* is the observation of *jth* individual, son of *ith* sire, with *k* age; *μ* is the general mean of the RFI; *βi* is the sire effect; *βj* is the age effect and *eij* is the random residual error, ~NID (0, *σ*^*2*^_*e*_); and *σ*^*2*^_*e*_ is the residual variance. The phenotypic measures included: initial body weight (BWi), final body weight (BWF), dry matter intake (DMI), average daily gain (ADG), feed conversion ratio (FCR), residual feed intake (RFI), residual body weight gain (RWG), residual intake and body weight gain (RIG), initial ribeye area (REAi), final ribeye area (REAf) and gain of ribeye area (REAg). Normality of data was tested by the Shapiro-Wilk test. Student’s t-test was applied to compare the groups for normally distributed variables and Mann-Whitney-Wilcoxon test for nonparametric variables using R STATS package. Results were considered significant when *p-value* (*P*) ≤ 0.05. The RFI values were adjusted using regression model, in which the age was fitted as a covariate for network analysis (Additional file 1).

### Sample collection

Serum samples were collected 21 days before the start of the feeding trial (before the adaptation period) by jugular venipuncture using vacutainer tubes. After 30 min at room temperature for clot formation, all samples were centrifuged at 3500×g for 15 min at 4 °C and stored at − 80 °C until further analysis, following the recommendations of Tuck et al. [24].

### LC-MS analysis

Protein precipitation of serum samples was performed at 4 °C by adding methanol (1:4 serum: methanol) and vortexing for 120 s at 5000 rpm [10]. The samples were then centrifuged at 16000 *g* for 4 min at room temperature, and the supernatants were dried in a vacuum centrifugal evaporator for 3 h at 30 °C and stored at − 20 °C prior to analysis. The samples were reconstituted in 200 μL H_2_O and centrifuged at 12000 rpm for 15 min. The supernatants were transferred to analytical vials for analysis using a Xevo G2 XS quadrupole-time-of-flight mass spectrometer (Q-TOF-MS) in positive and negative modes (Waters Corporation, Milford, MA, USA). Chromatographic separation was performed by an Acquity I-Class UPLC system (Waters Corporation, Milford, MA, USA) using a Waters Acquity BEH C18 column (2.1 mm × 100 mm, 1.7 μm) (Waters Corporation, Milford, MA, USA) at 50 °C. The injected sample volume was 5 μL. The mobile phase consisted of 0.1% formic acid-water (eluent A) and 0.1% formic acid-methanol (eluent B). The gradient elution in positive mode was performed at a flow rate of 0.4 ml/min, as follows: between 0 and 1 min 0% eluent B; 1–16 min increasing up to 100% eluent B;16–20 min at 100% eluent B and 20–22 min decreasing back to 0% eluent B. The elution flow rate was 0.36 ml/min in negative mode, with an elution gradient as follows: 0–2 min 0% eluent B; 2–17 min increasing up to 100% eluent B; 17–22 min at 100% eluent B and 22–24 min decreasing back to 0% eluent B.

The UPLC was connected to the electrospray ionization (ESI) interface, operating in negative and positive modes, with a capillary voltage of − 2.5/+ 3 KV, source temperature of 150 °C, cone gas flow of 50 L/h, cone voltage of 40 V, desolvation temperature of 550 °C and desolvation gas flow of 800 L/h. The spectra were collected at high resolution (mass resolving power 30,000 M/*Δ*M at fwhm) from 100 m/z (mass/charge ratio) to 1200 m/z, collected over 250 ms per spectrum in centroid mode. To avoid problems due to instrument drift, the sequence of samples was randomized and pooled quality-control samples (QC) were injected periodically for use in downstream data processing and correction [10]. QC samples were prepared by pooling equal volumes of all samples; these samples were run after every four sample injections to provide a measurement of the stability and performance of the system.

### Data treatment and pre-processing

LC-MS raw data were created and processed and using Waters MassLynx™ (Waters Corporation, Milford, MA, USA) Software v4.1 and Progenesis QI (Nonlinear Dynamics, Newcastle, UK). Following the manufacturer’s instructions, a reference run was automatically selected, and the precursor ion traces were processed for alignment, peak picking and normalization with default parameters. Locally estimated scatterplot smoothing (LOESS) signal correction based on QC samples was performed using MATLAB 2016 software with a script built for this purpose [25].

Afterward, a quality assurance (QA) step was used for analytical validation: variables with unacceptable reproducibility in QC samples (RSD > 20% in QCs or detected in less than 50% of QCs) and samples (detected in less than 90% of QC) were removed from the dataset [10]. The confidence scores of annotated metabolites are 2, meaning they have matches to a search database [26].

### Metabolomics data analysis

Univariate and multivariate analyses were carried out using Metaboanalyst 4.0 Web Server [27]. Glog transformation [28] and auto-scaling [29] were applied. Differences between the groups were investigated using univariate (UA) and multivariate analysis (MA). For MA, principal component analysis (PCA) and partial least-square discriminant analysis (PLS-DA) were used for detection of outliers and to identify features potentially responsible for variation between the groups [29]. PLS-DA model quality was assessed using the goodness of fit (R^2^) and goodness of prediction (Q^2^) in cross-validation and using a permutation test with 2000 permutations [29]. For UA, t-test was used to identify differentially expressed features, then the *p*-values were corrected for multiple tests by Significance Analysis of Microarrays (SAM-FDR) method [30]. Features with SAM-FDR *q-value* < 0.05 were considered different between groups.

### Network analysis

Network and clustering analysis were performed using the Weighted Gene Co-expression Network Analysis (WGCNA) R package [31, 32]. Normalized data from positive and negative acquisition modes were used separately as described by Fukushima et al. [33], with a soft threshold of 3, chosen using a scale-free topology criterion (R^2^ = 0.9). Modules containing at least 20 features were retained.

To select modules associated with FE, Pearson correlations between each module’s “eigengene” and the RFI were calculated. The “eigengene” is the first principal component of a given module and a representative measure of its metabolic profile. (The term “gene” is used even for other data types, due to the development of WGCNA originally for the analysis of transcriptional data.) Modules with a module-trait relationship magnitude (correlation) > 0.5 for RFI (*P* ≤ 0.05) were considered significant. Individual features were considered for further analysis only if they had module membership (MM) > 0.6 (*P* < 0.01) and gene significance (GS) > 0.5 (*P* < 0.05). GS is defined as the association of features with RFI, and MM is defined as the correlation of the features with the module eigengene. High GS and MM scores indicate a feature is a central element of a module and is significantly associated with the trait [34].

### Metabolic pathway analysis

Metabolic pathway analysis was performed using Mummichog software 1.0.9 with *Bos taurus* species (KEGG database) as reference [35]. Using default parameters for analyte prediction (mass accuracy 10 ppm) and for pathway enrichment analysis (1000 permutations). Features from UA with *P* < 0.01 were used as input to mummichog to test for pathway enrichment compared to random data resampled from the reference list, yielding an empirical *p-value* per pathway. Pathways with corrected *q-value* < 0.05 were considered significant.

## Results

We performed a 70-day feeding trial on 98 Nellore young bulls to evaluate their feed efficiency [9]. Based on the linear model (see Methods), there was no significant sire effect on RFI and the high feed efficient (HFE) and the low feed efficient (LFE) groups were statistically different (*P* ≤ 0.05) for all FE traits (feed conversion ratio (FCR), RFI, residual weight gain (RWG) and residual intake and weight gain (RIG), dry matter intake (DMI)) and also for average daily gain (ADG). There was also a significant difference for backfat thickness at the end of the experiment (BFTf, *P* ≤ 0.05), which were greater in the LFE group (Table 1). Therefore, HFE animals in this experiment are more sustainable since they eat less, are leaner and have a better ADG than LFE animals.

**Table 1:** Descriptive statistics of high feed efficiency (HFE) and low feed efficiency (LFE) for phenotypic traits

Trait	HFE (±SEM)	LFE (±SEM)	*P* value
BWi (kg) ■	410 ± 16.03	404.3 ± 7.97	0.64
BWf (kg) ○	563.5 ± 17.35	525.8 ± 9.87	0.07
DMI (kg/d) ■	10.38 ± 0.39	12.35 ± 0.33	< 0.0001*
ADG (kg/d) ■	2.194 ± 0.15	1.734 ± 0.08	0.0497*
FCR ■	4.763 ± 0.17	7.3 ± 0.29	< 0.0001*
RFI (kg/d) ○	−1.384 ± 0.12	1.791 ± 0.12	< 0.0001*
RWG (kg/d) ■	0.4325 ± 0.07	− 0.3988 ± 0.06	< 0.0001*
RIG ○	1.815 ± 0.10	−2.188 ± 0.13	< 0.0001*
REAi (cm2) ■	68.26 ± 2.22	67.23 ± 1.95	0.63
REAf (cm2) ■	84.94 ± 2.58	82.91 ± 1.65	0.64
REAg (cm2) ■	19.34 ± 2.83	15.69 ± 1.77	0.99
BFTi (mm) ○	0.775 ± 0.38	1.975 ± 0.46	0.07
BFTf (mm) ■	2.975 ± 0.67	5.713 ± 0.64	0.0096*
BFTg (mm) ■	2.2 ± 0.67	3.738 ± 0.37	0.063

*BWi* initial body weight, *BWF* final body weight, *DMI* dry matter intake, *ADG* average daily gain, *FCR* feed conversion ratio, *RFI* residual feed intake, *RWG* residual body weight gain, *RIG* residual intake and body weight gain, *REAi* initial ribeye area, *REAf* final ribeye area, *REAg* gain of ribeye area. *P < 0.05. ■ Student’s t-test. ○Mann-Whitney-Wilcoxon Test

### Metabolome profile and differential analysis

**Fig. 1:**
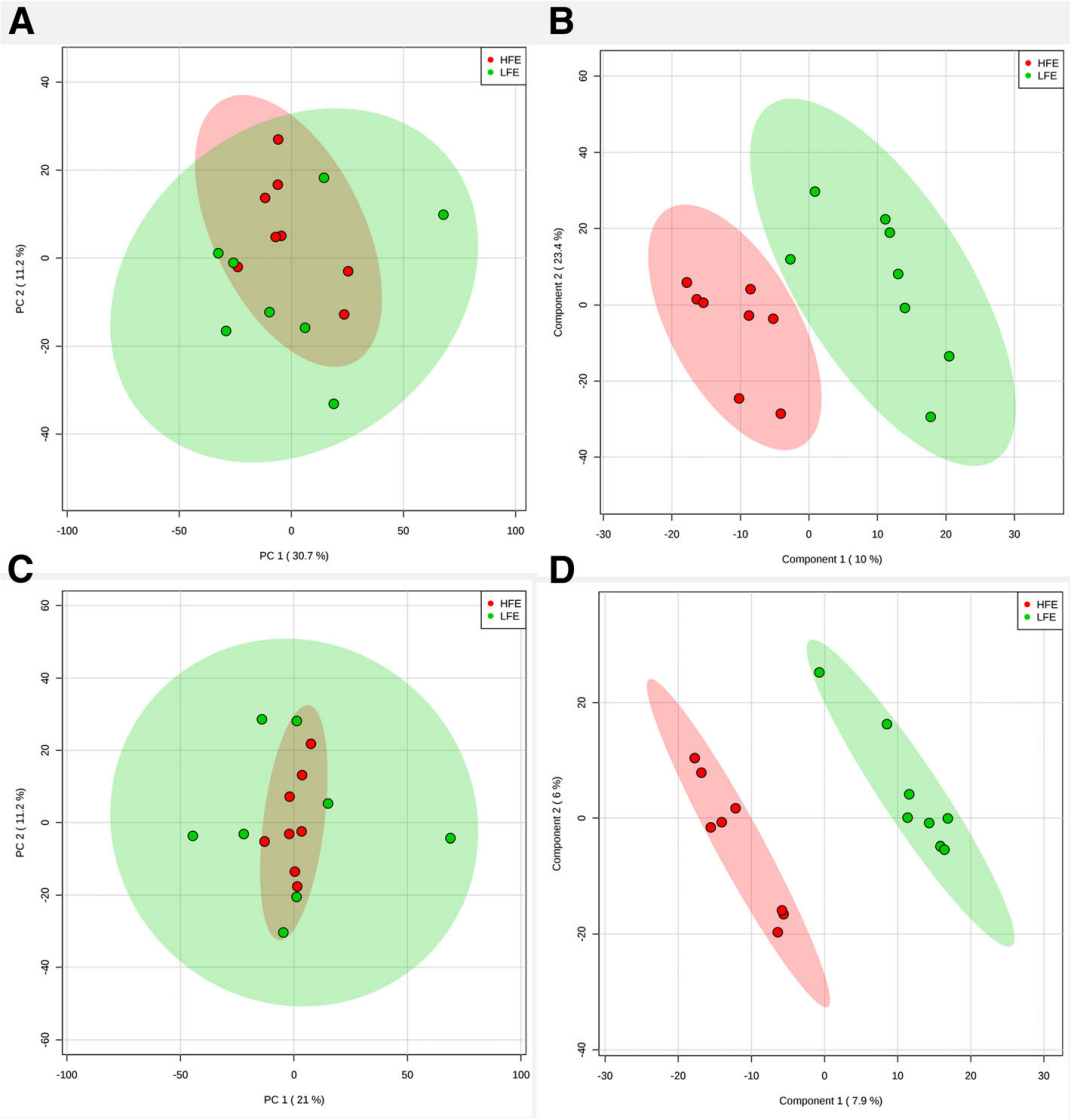
PCA (**a** and **c**, in negative and positive mode, respectively) and PLS-DA (**b** and **d**, negative and positive mode, respectively) scores plots based on LC/MS data of serum samples from HFE (red) and LFE (green). The PLS-DA models discriminated between HFE and LFE groups (R^2^ of 0.87 and 0.98 in negative and positive mode, respectively) but were not predictive (Q^2^ of 0.08 and 0.15). Considering a common heuristic for metabolomics data: R^2^ > 0.8 and Q^2^ > 0.5, the model was not overfitted. Consistent with this, a permutation test (2000 permutations) yielded *P*-values > 0.9 in both modes

**Fig. 2:**
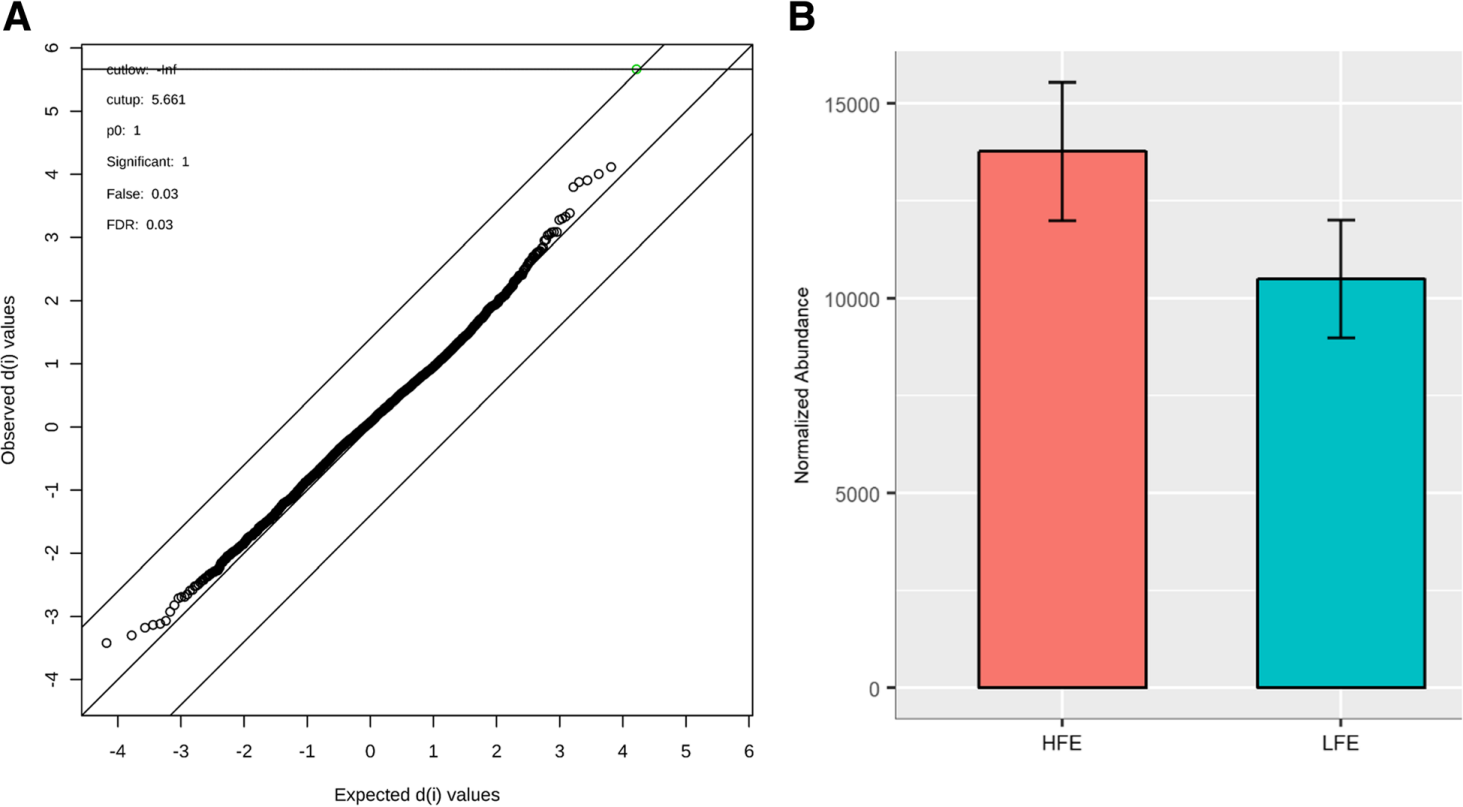
Univariate differential analysis of features from bovine metabolome. **a** Univariate analysis corrected by multiple tests (SAM-FDR) results for positive mode features. **b** The difference of abundance between the HFE and LFE groups for the m/z 183.1670 peak with a retention time of 4.00 min (positive mode; SAM-*FDR* ≤ 0.05)

After quality assurance processing, a total of 3598 and 4210 m/z in negative and positive ionization modes, respectively, were used for parallel analyses. For Principal component analysis (PCA), no separation was observed for high and low FE animals in the first five principal components (Fig. 1), which explained 64.5 and 57% of total variance for negative and positive modes, respectively. PLS-DA was able to distinguish the two groups, but permutation and cross-validation analyses indicated the model was overfitted and thus not predictive (Fig. 1). The univariate analysis yielded one feature with different abundance between groups in positive mode. The spectra of mass-charge 183.1670 m/z and retention time v4.00 min on chromatography column (Fig. 2) has a *P* < 0.001 (SAM-FDR = 0.03) which is greater on HFE group. No significantly different m/z were identified in negative mode.

### Pathway enrichment analysis

Pathway enrichment analysis was performed to explore possible pathways involved in RFI phenotypic variation prior to the feedlot. Mummichog software identified the enrichment of retinol metabolic pathway (*P* < 0.05; Table 2), as being associated with FE in positive mode with 2 pathway metabolites annotated in the data. The putative compounds hit included retinoate (C00777) and, either the isobaric compounds (molecular weight 284.4357): all-trans-Retinal (C00376) or 11-cis-Retinal (C02110) (Table 3).

**Table 2:** Metabolic pathways for RFI prior to the feedlot and their size on the positive mode of acquisition

Pathway	Pathway size	Total Hits	Significant Hits	Fisher’s *P* value
Retinol metabolism	17	6	2	0.0237*
Steroid hormone biosynthesis	67	8	1	0.3055
Arachidonic acid metabolism	36	7	1	0.2725

**Table 3:** Significant analytes predicted by mummichog

m/z	Compound	adduct	mass diff	*P* value	HFE/LFE
267.2105	all-trans-Retinal / 11-cis-Retinal	M-H2O + H[1+]	0.00017686	0.0075*	Down
273.2233	Retinoate	M-CO + H[1+]	0.00213163	0.0019*	Up

The mass-charge (m/z), compounds hit, mass difference, analyte *p*-value and FE group association

### Weighted correlation network analysis

We then used WGCNA co-expression analysis to identify clusters of analytes that may have a relationship with the feed efficiency. WGCNA identified 19 and 20 modules of highly correlated features in negative and positive mode, respectively.

**Fig. 3:**
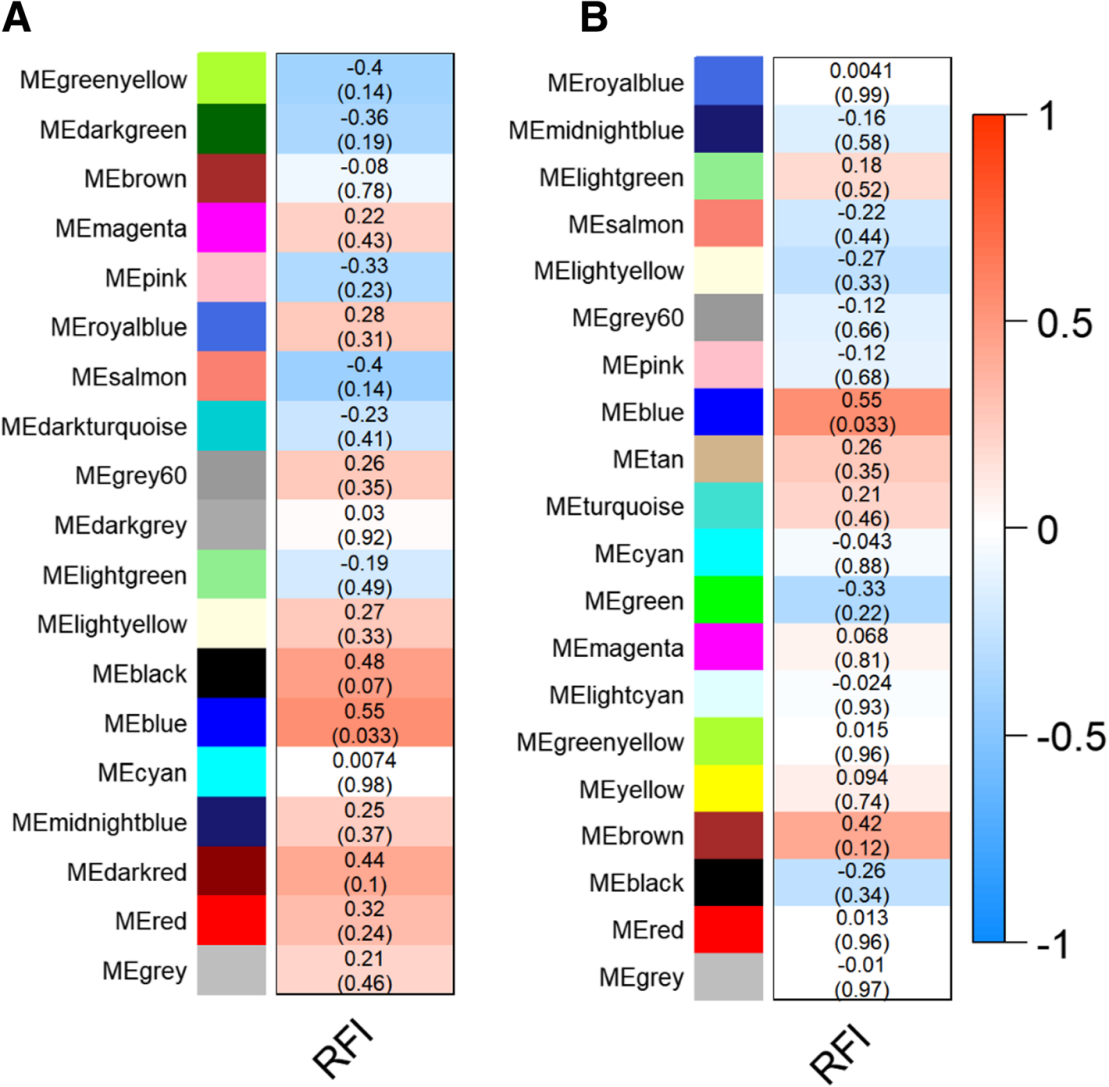
Network analysis of co-expressed features in the negative and positive mode of acquisition. Pearson correlation between residual feed intake (RFI) and the module eigengenes in the negative (**a**) and positive (**b**) mode. In each line the color name of modules (ME). The number in each module is the Pearson correlation between the module and RFI; In brackets the *p*-value of the correlation

One of these modules was significantly positively correlated with RFI (blue module from the negative mode, *r* = 0.55, and *P* = 0.033), indicating higher levels in LFE animals. The blue module contains 196 features (Fig. 3a), of which 65 were identified as important contributors to this module (Additional file 2). Using mummichog, three of these features were putatively annotated: (i) 6S,9R-Vomifoliol (compound KEGG C01760) (ii) 2,3, Dihydroflavone (compound C00766); (iii) Limonoate (compound C01593). The additional file 2 has information of the important features of blue module on negative mode, including mass-charge (m/z), retention time (rt), feature significance to trait (GS), feature significance to module (FM), group with highest abundance, putative matched compound in KEGG and mass difference between feature and putative compound.

A second module was associated with RFI (blue module from the positive mode, r = 0.55, and *p-value* = 0.033), also indicating higher levels in LFE animals. This module contains 112 features (Fig. 3b), of which 39 were identified as highly important contributors to this module (Additional file 3). Using mummichog, 5 compounds were annotated: (i) Phytanic acid (compound C01607); (ii) all-trans-Retinal (compound C00376); (iii) Progesterone (compound C00410); (iv) Limonoate (compound C01593); (v) Stearic acid (compound C01530). The additional file 3 has information of the important features of blue module on positive mode, including mass-charge (m/z), retention time (rt), feature significance to trait (GS), feature significance to module (FM), group with highest abundance, putative matched compound in KEGG and mass difference between feature and putative compound.

## Discussion

Brazilian cattle are mainly raised in pastures, but can also be kept in feedlot systems with diets composed of silage or other feedstuffs, such as high-fiber crop residues or grains (corn and soybean), to improve body weight gain before slaughter. We used serum samples collected before the feedlot period to search for a potential early metabolomic signature for FE, with the intent to support nutritional management decisions to improve productivity and sustainability of livestock. Thus, we performed an exploratory analysis using untargeted metabolomics coupled with bioinformatics and interpretation tools including Mummichog and WGCNA. We found one differentially expressed feature between HFE and LFE animals in these conditions, but most importantly, we also found one enriched pathway and two sets of highly correlated features significantly associated with FE, which could be considered a potential molecular signature of FE in Nellore cattle before they enter the feedlot period.

A co-expression module associated with a phenotype provides significant promise for the development of a molecular signature, clearly more than a single statistically different feature between two conditions [36]. In our previous work, the co-expression gene modules and their gene ontology were far more important results than the differentially expressed genes [9]. In this context, the hepatic inflammatory response was associated with feed efficiency in cattle. Here, the WGCNA analysis indicated two modules of co-expressed features positively associated with RFI, with equal correlation, *p*-values (Fig. 3) and common features, suggesting that both networks belong to the same molecular signature.

We were able to identify 7 molecules from the co-expressed modules through Mummichog prediction: Retinal, Progesterone, Stearic acid, Vomifoliol, 2,3 Dihydroflavone, Limonoate and Phytanic acid. Interestingly, all these molecules have higher levels in LFE animals which are in accordance with the modules being positively associated with RFI. In addition, mummichog software predicted two molecules from the retinol pathway significantly associated with FE: a higher level of Retinal and lower level of Retinoate (C00777) in LFE, which implies the enzymes aldehyde oxidase and retinal dehydrogenase (that convert Retinal to Retinoate) as probably less active/expressed in LFE animals. This result is in accordance with Zhao and colleagues [37] who demonstrate vitamin A (VA) metabolism is important for feed efficiency in pigs as key genes of VA metabolism such as ALDH1A2 and CYP1A1 are upregulated in the liver of HFE animals. Also, in two transcriptome studies, the retinol pathway was upregulated in the liver of high-RFI Jersey steers [38] and over-represented in the small intestine from high intake beef steers [39]. A GWAS-study using CNV markers evidenced the RDH5 (an important gene of the retinol metabolism pathway) as a candidate gene associated with feed conversion rate in Nellore cattle [40]. Therefore, our results agreed with the literature regarding the importance of the retinol metabolism pathway for feed efficiency in livestock animals.

Progesterone (P4) was another feature predicted in the molecular signature of FE being more present in the blood of LFE animals. Steroid hormone biosynthesis was overrepresented in the set of genes in the liver that were upregulated in the high-RFI (low FE) group of Jersey cows [38], which is in accordance with our results. Recently, P4 signaling in broiler skeletal muscle was associated with divergent feed efficiency [41]. So far, there is no consensus on the role of P4 on feed efficiency in livestock and further studies should be performed.

The stearic acid is a saturated acid (C18:0) and one of the end products of the fatty acid biosynthesis pathway in animals. This fatty acid was found increased in plasma of steers with least ADG in comparison with greatest ADG [42], and this result corroborates our finding of a higher level of stearic acid in the molecular signature associated with LFE animals since they had less ADG than HFE in our experiment.

From all predicted molecules, Vomifoliol, 2,3 Dihydroflavone, Limonoate and Phytanic acid are molecules produced exclusively by bacteria or plants and not mammals. The higher presence of these molecules in the blood of LFE animals could be due to higher DMI of these animals in comparison with HFE animals, allowing the higher presence of these metabolites in the blood. However, this possibility lacks further evidence since we did not evaluate the pasture DMI of these animals, i.e. feed intake before they arrive at the feedlot. From these 4 molecules, the Phytanic Acid could have a role on feed efficiency. Phytanic acid is a branched-chain fatty acid formed during the metabolism of phytol [43] by ruminal bacteria and is a known agonist for the nuclear-receptor-retinoid-X-receptor [44] and the peroxisome proliferated-activated receptor-α (PPAR-α) [45]. These two proteins are important nuclear receptors regulating the expression of several genes in response to environmental factors (i.e. diet) and endogenous molecules. Interestingly, in rats, agonists of PPAR-α decreased feed efficiency [46], and the PPAR signalling pathway was enriched in the small intestine transcriptome analysis of high vs. low feed intake cattle [39]. Therefore, agonists of PPAR-α could reasonably be associated with feed efficiency in cattle, but new evidence should be provided to confirm this hypothesis.

Our integrated approach using data annotation, mummichog prediction and WGCNA co-expression analyses indicated a molecular signature enriched for biological processes previously associated with FE. The metabolites in WGCNA modules were also predicted by mummichog, which supports the validity of the in silico network analysis since the two different analyses yielded consistent results. Therefore, we believe metabolomics based modules associated with FE possibly represent a molecular metabolic signature of FE. Although we have not yet been able to identify the majority of the features in those modules, previous studies on feed efficiency support the network analysis results. Moreover, we noted it is possible to have a molecular signature associated with a phenotype without knowing the function of the components, just by (for metabolites) tracking m/z ratio and retention time in a standardized assay. As an example, this is the case for commercially available genomic selection in dairy cattle using DNA markers, where the majority of the markers are not functional SNPs.

In our data, we found only one feature statistically different between the FE groups: the feature with m/z 183.1670 and RT of 4.00 min (positive mode) is upregulated in HFE animals. This result along with the co-expressed module provides evidence of early serum metabolome differences between high and low FE animals. Between both the positive and negative ionization modes and after quality control-based filtering, the serum metabolome of the animals in this experiment consisted of approximately 8000 features. One may expect a priori to identify more than just one different feature between high and low FE animals using such a powerful tool. Possible explanations for this result include, but are not limited to: (1) although the groups are very distinct phenotypically at the end of the experiment, their baseline metabolic profiles may have been more similar at the time when samples were collected (21 days before the beginning of the feeding trial) [9]; (2) the FE was estimated for feedlot performance and not for pasture grazing; at the time of sampling all animals were still on pasture conditions, which may yield more similar metabolic phenotypes than a high grain diet; (3) the animals were clinically healthy over the whole experiment. Thus, no major physiological disturb could lead to large metabolome difference between the FE groups; (4) the number of sampled animals (8 animals per group) could limit the statistical power [47] for these outbred, genetically different animals that may have high baseline diversity in metabolic profiles. To address this last issue, one of our ongoing projects is to validate these results in a cohort with more animals, to develop a future technology help establish a framework for future for FE prediction.

## Conclusion

The conclusion from this work is the detection of a molecular signature for feed efficiency of beef cattle based on untargeted metabolomics. This molecular signature indicated the vitamin A metabolism pathway as one of the important pathways for this phenotype.

## Additional files

Additional file 1: Experiment information of animals including group, birth, days of life at before feedlot (− 21 days), father, residual feed intake and residual feed intake adjusted by age as a covariate. The FE groups had different ages (*P* < 0.05). To perform the Network analysis, the phenotype was adjusted by age, fitted as a covariate. (CSV 905 bytes)

Additional file 2: Important features in blue module in negative acquisition mode. Mass/charge ratio (m/z); Gene significance (GS); Module Membership (MM); Feature connectivity within the module (Kwithin); Adducts; Highest abundance group; Adducts; Matched Compound (KEGG by mummichog); Mass difference between m/z and matched compound. (CSV 6 kb)

Additional file 3: Important features in blue module in positive acquisition mode. Mass/charge ratio (m/z); Gene significance (GS); Module Membership (MM); Feature connectivity within the module (Kwithin); Adducts; Highest abundance group; Adducts; Matched Compound (KEGG by mummichog); Mass difference between m/z and matched compound. (CSV 3 kb)

## Abbreviations

ADG: Average daily gain
ALDH1A2: Aldehyde Dehydrogenase 1 Family Member A2 gene
CNV: Copy-number variation
CYP1A1: Cytochrome P450 Family 1 Subfamily A Member 1 gene
DMI: Dry matter intake
FE: Feed efficiency
HFE: High feed efficiency group
LFE: Low feed efficiency group
NEG: Negative mode acquisition
P4: Progesterone
POS: Positive mode acquisition
PPARα: Peroxisome proliferated-activated receptor-alpha
RDH5: 11-cis retinol dehydrogenase 5 gene
RFI: Residual feed intake
VA: Vitamin A
